# Cholera and COVID-19 pandemic prevention in multiple hotspot districts of Uganda: vaccine coverage, adverse events following immunization and WASH conditions survey

**DOI:** 10.1186/s12879-023-08462-y

**Published:** 2023-07-21

**Authors:** Godfrey Bwire, Annet Kisakye, Esther Amulen, John Baptist Bwanika, Joan Badebye, Christine Aanyu, Brenda Doreen Nakirya, Alfred Okello, Stephen Acellam Okello, Justine N. Bukenya, Christopher Garimoi Orach

**Affiliations:** 1 School of Public Health, Makerere University, Kampala, Uganda; 2 Division of Public Health Emergency Preparedness and Response, Ministry of Health, Kampala, Uganda; 3 World Health Organization, Kampala, Uganda; 4 School of Forestry, Environmental and Geographical Sciences, Makerere University, Kampala, Uganda; 5 Community Concerns Uganda Initiative, Jinja, Uganda; 6 Department of Public Health, St Mary’s Hospital Lacor, Gulu, Uganda; 7 Department of Health Services, Kyambogo University, Kampala, Uganda

**Keywords:** COVID, Vaccine hesitancy, Cholera, Africa, Pandemic, Uganda, Water coverage, Sanitation, Vaccine coverage, WASH, Adverse event following immunization, Coronavirus

## Abstract

**Background:**

Between March, 2020 and December, 2021 due to cholera and coronavirus disease 2019 (COVID-19) pandemics, there were 1,534 cholera cases with 14 deaths and 136,065 COVID-19 cases with 3,285 deaths reported respectively in Uganda. This study investigated mass vaccination campaigns for the prevention of the two pandemics namely: oral cholera vaccine (OCV) and COVID-19 vaccine coverage; adverse events following immunization (AEFI); barriers and enablers for the vaccine uptake and assessed water, sanitation and hygiene (WASH) conditions in the six cholera and COVID-19 hotspot districts of Uganda.

**Methods:**

A household survey was conducted between January and February, 2022 in the six cholera hotspot districts of Uganda which had recently conducted OCV mass vaccination campaigns and had ongoing COVID-19 mass vaccination campaigns. The survey randomly enrolled 900 households with 4,315 persons of whom 2,085 were above 18 years. Data were collected using a data entry application designed in KoBoToolbox and analysed using STATA version 14. Frequencies, percentages, odds ratios, means, confidence intervals and maps were generated and interpreted.

**Results:**

The OCV coverage for dose one and two were 85% (95% CI: 84.2—86.4) and 67% (95% CI: 65.6—68.4) respectively. Among the 4,315 OCV recipients, 2% reported mild AEFI, 0.16% reported moderate AEFI and none reported severe AEFI. The COVID-19 vaccination coverage for dose one and two were 69.8% (95% CI: 67.8–71.8) and 18.8% (95% CI: 17.1–20.5) respectively. Approximately, 23% (478/2,085) of COVID-19 vaccine recipient reported AEFI; most 94% were mild, 0.6% were moderate and 2 cases were severe. The commonest reason for missing COVID-19 vaccine was fear of the side effects. For most districts (5/6), sanitation (latrine/toilet) coverage were low at 7.4%—37.4%.

**Conclusion:**

There is high OCV coverage but low COVID-19 vaccine and sanitation coverage with high number of moderate cases of AEFI recorded due to COVID-19 vaccines. The low COVID-19 vaccine coverage could indicate vaccine hesitancy for COVID-19 vaccines. Furthermore, incorporation of WASH conditions assessment in the OCV coverage surveys is recommended for similar settings to generate data for better planning. However, more studies are required on COVID-19 vaccine hesitancy.

**Supplementary Information:**

The online version contains supplementary material available at 10.1186/s12879-023-08462-y.

## Background

Cholera and Coronavirus disease 2019 (COVID-19) are the two ongoing devastating pandemics in sub-Saharan Africa [1, 2] and have a common origin in Asian continent [3, 4]. Cholera is a diarrheal disease caused by a bacteria *Vibrio cholerae* [5]. It is an old disease, with seven recorded pandemics [6]. The seventh cholera pandemic started in 1961, in Indonesia and is still being considered by the World Health Organization (WHO) as an ongoing pandemic [7]. In developed countries, cholera was eliminated over a half a century ago [8]. However, cholera remains a big threat causing many deaths especially in sub-Saharan Africa and some parts of Asian continent [9]. In 2021, 90 countries reported a total of 223,370 cholera cases and 4,159 deaths [10]. Cholera is transmitted between humans through faeco-oral route [11]. Prevention of cholera is by universal access to safe water, sanitation and hygiene (WASH) [12]. For those areas with inadequate WASH, use of oral cholera vaccine (OCV) is an important intervention to complement WASH, surveillance and case management in prevention of the cholera pandemic [13, 14]. There are three WHO pre-qualified OCVs namely, *Dukoral®, Shanchol™ and Euvichol-Plus®* [15]. In order to confer full protection by OCV, the person should receive two doses of a particular vaccine that are separated by at least 14 days apart [16].

On the other hand, COVID-19 is a recent disease which was first reported in Wuhan, China in December 2019 [3]. It soon spread to other parts of the world and in March 2020, COVID-19 was declared a pandemic [17]. COVID-19 is a respiratory viral disease that is caused by severe acute respiratory syndrome coronavirus 2 (SARS-CoV-2) [3]. The data on the global reported COVID-19 cases and deaths for the period from 31^st^ December 2019 – 31^st^ December 2022, showed that COVID-19 affected developed countries and continents more than developing countries in Africa [18, 19]. As of 31^st^ December 2022, over 761 million cases and more than 6.8 million deaths due to COVID-19 were confirmed and reported to the WHO [18]. Transmission of SARS-CoV-2 can be by contact, droplet, airborne, fomite, fecal–oral, bloodborne, mother-to-child, animal-to-human and human-to-human as occur when an infected person coughs, sneezes, talks or sings [20, 21]. Moreover, just like cholera where there are asymptomatic or mild cases [22, 23], majority of the COVID-19 cases have mild symptoms [24]. In few of the COVID-19 cases, the disease is more severe, requiring admission, oxygen therapy and intensive care [25]. Moderate and severe COVID-19 cases require hospitalization and appropriate treatment [26]. Prevention of COVID-19 spread is through vaccination, isolation of confirmed cases, hand-washing, wearing of masks, social distancing, quarantine and lockdown [24, 26–28]. Mass vaccination through the use of the WHO approved vaccines is an effective way to prevent COVID-19 [28]. SARS-CoV-2 has many variants of the original virus which keep on evolving [29]. Notable among the variants are *Alpha, beta, delta and Omicron* [30–32].

While COVID-19 pandemic is a recent disease in Africa [19], the seventh cholera pandemic reached Africa in early 1970s and is endemic in sub-Saharan Africa [6, 33]. Cholera is endemic in Uganda with the records going back to over a half a century [34]. COVID-19 was first confirmed in Uganda on 21^st^ March, 2020 [35]. Both cholera and COVID-19 pandemics can be prevented using similar approaches such as hand-washing and hygiene, social distancing, restriction of large gatherings, improved general hygiene in health facilities and households, lockdown and vaccination with appropriate vaccines [16, 36–38].

Prevention of cholera by mass vaccination confers herd immunity to the community when coverage of above 50% with two doses of a particular OCV is achieved [38]. For COVID-19 pandemic, vaccination coverage of at least 70% coverage of the target population of persons aged 18 years and above with two doses of the two dose vaccine is required [39]. Consequently, in order to effectively monitor vaccination programs and to establish if the protective coverages have been achieved often coverage surveys are carried out [40]. It should be noted that though OCV is effective in preventing cholera epidemics, this prevention is not permanent [38]. Often, protection with OCV lasts 3–5 years. Hence, to sustain the benefits of OCV if no improvement in WASH has been recorded in the subsequent years after the campaigns, repeat OCV mass campaigns are recommended [41].

In 2017, the WHO launched a global cholera elimination roadmap that has a target to eliminate cholera by 2030 in some partner states [14]. In this roadmap, a number of interventions to reduce cholera were recommended and they included mass vaccination of the communities in cholera hotspot districts [42]. In 2017, the government of Uganda developed a five year plan [43] to guide cholera preventive activities. This plan had interventions recommended in the WHO global cholera elimination roadmap [14] such as mass vaccination with OCV for cholera hotspot districts. In 2018, Uganda applied for OCV from the World Health Organization, Global Taskforce for Cholera Control (GTFCC) and carried out a series of OCV campaigns which continued to the end of 2021. In March, 2020 the first case of COVID-19 was confirmed in Uganda and thereafter the two pandemics remained major public health issues requiring strong interventions to mitigate them. Between March, 2020 and December, 2021 at least 1,534 cases with 14 deaths of cholera [44] and 136,065 cases with 3,285 deaths of COVID-19 [45] were reported respectively. Mass vaccination using WHO recommended vaccines [46, 47] are some of the interventions that the Uganda government uses to prevent these pandemics. COVID-19 vaccines are given intramuscularly and like OCV, commonly two doses (except *Jonson and Johnson vaccine where a single dose is adequate*) are administered for full protection. The doses are separated by a period of not less than 21–28 days. In Uganda, the following COVID-19 vaccines have been in use namely, *Astra Zeneca, Moderna, Sinovac, Sinopharm, Pfizer and Jonson and Johnson.* The OCV that has been used since 2018 in Uganda is *Euvichol plus* (Eubiologics, Korea). However, the vaccines are known to cause mild to moderate side effects that resolve within a few days [40, 48] but these can negatively affect vaccine uptake by the communities. To ensure high COVID-19 vaccine uptake, the government of Uganda collaborates with several partners including the WHO Kampala office, the Irish and Norwegian governments, among others [49].

On the other hand, adequate WASH services is the ultimate solution for prevention of cholera outbreaks [38] and a pillar for healthy living [50]. However, often after the OCV campaigns the coverage surveys are done [40, 51–53] without WASH assessments which are equally important to plan for the long-term (permanent) cholera prevention. Therefore, the objectives of this study were to: (i) estimate the OCV and COVID-19 vaccination coverage, (ii) estimate the frequency of AEFI that occurred during and after the vaccination campaigns, (iii) analyze barriers and enablers of vaccine uptake and (iv) assess household's WASH conditions.

## Methods

### Study design

This was a cross-sectional study in which quantitative data were collected by survey methods and analysed to describe status of service delivery in the study communities.

### Study setting

The survey was carried out in three out of the four regions of Uganda with districts that were previously categorized as cholera hotspots [42]. From each of the region, two districts that met the following criteria were selected.

1. Cholera hotspot districts.
2. Recently (month of the last activity implementation, September 2021) conducted OCV campaign.
3. Reporting COVID-19 new cases and deaths.
4. Carried out or had an ongoing COVID-19 vaccination campaign.

To decide if the selected district was experiencing COVID-19 epidemic, the investigators used weekly epidemiological reports of the Uganda Ministry of Health [44].

**Fig. 1:**
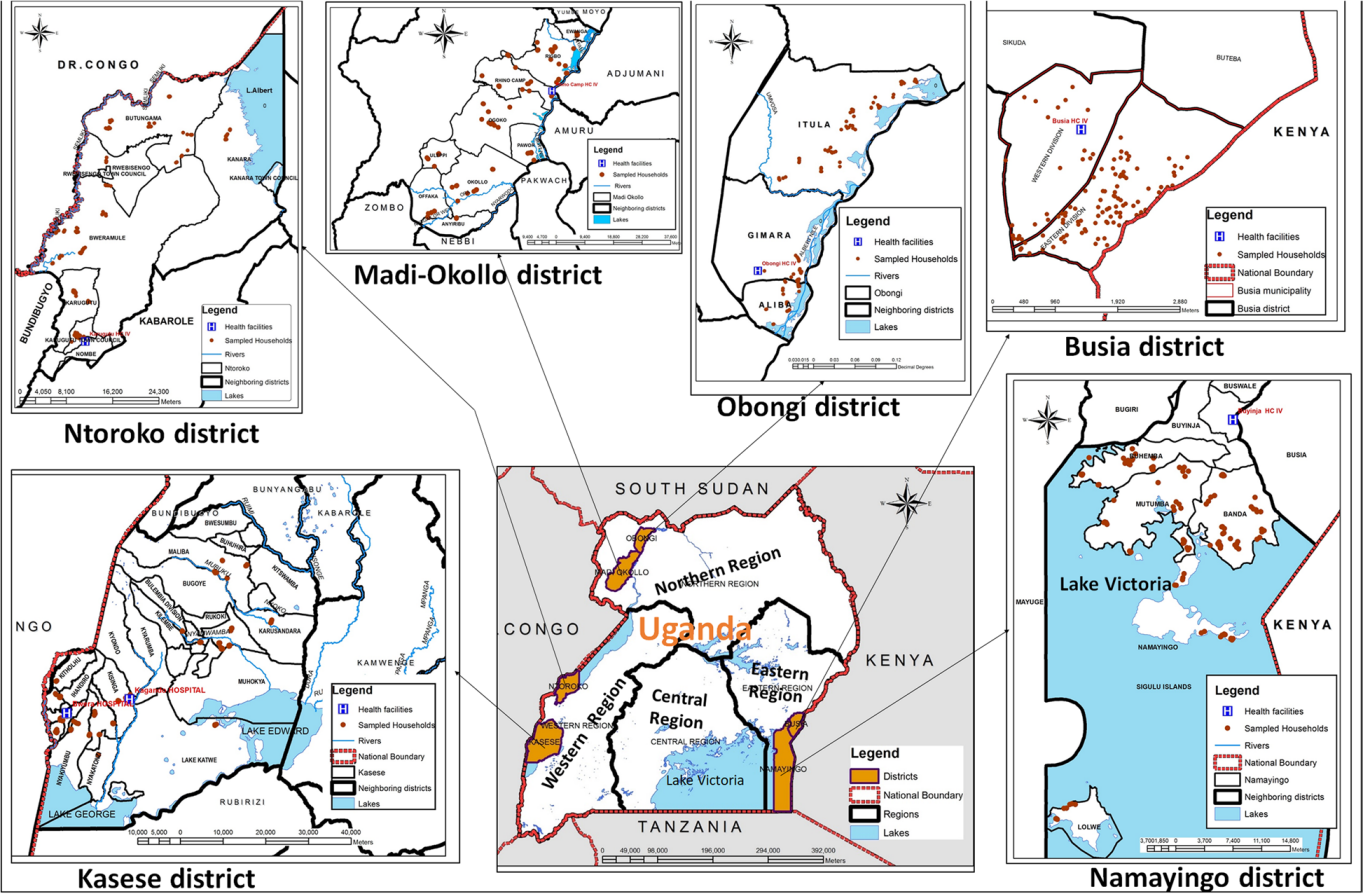
Map of Uganda showing the geographical regions and the locations of the study area. The map was created using Arcmap software, version 10.5 [54]. The brown dots represent the locations of sampled households. All the six districts are located along the international borders

In total, six districts of Obongi, Madi-Okollo, Busia, Namayingo, Ntoroko and Kasese were purposively selected. Selected districts were all located along the international country borders. The locations of the six districts in Uganda are shown in Fig. 1.

Data were collected by a team of 39 trained research assistants (RAs) who were supervised by a core team of 5 investigators (1 principal investigator and 4 co-investigators). The training of RAs and pretesting of the data collection tools were carried out from 10^th^ – 14^th^ January 2022. Thereafter, data were collected from 17^th^ – 29^th^ January 2022.

### Sample size determination

The sample size was calculated by applying the standard formula shown below [55]

$${\mathrm{N}}{.}=\frac{{\mathrm{z}}^{2}\mathrm{p}(1-\mathrm{p})}{{\mathrm{d}}^{2}}$$

where 

${\mathrm{N}}{.}$ is the number of individuals required in the survey

$\mathrm{z}$ is the normal deviate (1.96 for an alpha of 0.05)

$\mathrm{p}$ is the proportion of individual expected to be treated with diarrhoea at the health facility

$\mathrm{d}$ is the precision (acceptable error) of the estimate

The proportion of people who received two doses in the initial integrated OCV campaign in response to a cholera outbreak in Hoima district, Uganda (p) which was found to be 78% [40]. Also, a desired precision level of 10% with a design effect (DEFF; the difference between vaccination coverage inside the clusters compared to between them) of 3, a confidence level of 95% and anticipated non-response rate of 10% were applied. Hence, a total of 150 respondents per district were selected giving 900 respondents for the six study districts.

### Selection of the villages and respondents within the study districts

The survey was conducted following similar methodology, criteria and procedures as previously described [40]. However, there were modifications to cater for WASH conditions assessment which often are omitted during OCV coverage surveys. Thirty (30) clusters each consisting of 5 households were sampled from each of the six selected districts. The sampling frame consisted of all the villages in the districts where OCV was administered. A total of 30 villages were randomly selected from a frame of all villages in the district. The first task in household selection was to get sub-county population and proportionately distribute the 30 villages according to these population. Thus, the sub-counties that had bigger population were proportionately allocated more villages (clusters). Next, in each of the selected villages, survey field supervisors identified the village heads (Local councils I) who provided the registers of the inhabitants in the village. From each village register, five (5) households were randomly selected giving a total of 150 households. Within a household, the household head was identified and interviewed by use of two sets of pretested questionnaires. The first set of questionnaire collected information on OCV and COVID-19 vaccine coverage, AEFI, enablers and barriers to vaccination. The second set of a questionnaire and a checklist (WASH assessment tool) collected WASH specific data. WASH assessment tool used in this study is shown in the Additional file 1.

### Study variables

The study variables included:

Location of the house, number of persons who lived in the household, their ages and sex, evidence of receiving the vaccine by asking for and checking the vaccination card or getting convincing explanation on what was done. Also, for those who did not received the vaccine, the reason why the vaccine was not administered was inquired and noted. While listing the ages of the household members, the RAs excluded those who were found to be outside the target age-groups at the time when the vaccination campaign took place. The target group for OCV vaccination was one or more years [39] while that for COVID-19 was equal to 18 years or more as per Ministry of Health guidelines [56]. In order to simultaneously control COVID-19, MOH imposed special COVID-19 countermeasures throughout Uganda. Consequently, there was delayed administration of the second OCV dose in all the six districts in this study. Hence, to ensure that this delay in administration of the second OCV dose had minimal effect on the collected data, the RAs were equipped with techniques to fish-out homesteads which received OCV from those which did not. For instance, in the homes where the household head could not produce the card, the RAs asked and checked the house/home to see if it had a specific label that was used to mark the households that received the first dose OCV.

The variables to assess WASH included;

- *Household water:* Source of household water, time spent collecting water and treatment or boiling of drinking water.
- *Sanitation:* availability of latrine or toilet (includes latrine of all types but with washable floor, toilet and ecosan or any other recommended as safe), type of feacal disposal facility (latrine, toilet, ecosan and other appropriate one) and ownership of the latrine or toilet facility by the family.
- *Hygiene:* availability of hand-washing facility, availability of soap and for those that washed hands.

Variables to identify AEFI involved asking for AEFI for each individual member of the household who received vaccines. For those with AEFIs, what was done after noticing the AEFI (reported or not reported to the health workers).

### Categorization of adverse effects following immunization (AEFI)

The AEFI were categorized into three groups namely;

1. *Mild*: The person continued with the normal activities;
2. *Moderate*: The person was able to continue with most activities but the symptoms interfered somewhat and
3. *Severe*: The clinical symptoms interfered so much that the person was not able to continue with normal activities.

### Data management and analysis

Data were collected by trained RAs. The RAs were recruited from health workers who were familiar with the study districts in terms of culture and the languages spoken. Research assistants were trained on survey protocol and on the use of KoBoToolbox application [57] prior to field deployment. Data were collected and entered into a data entry application designed in KoBoToolbox [57] using mobile phones. This application was designed to avoid erroneous entries through proper validation of fields; to allow only the entry of the possible values for a field. Also, skip patterns and value labels were incorporated into the application. Data were routinely checked for errors and inconsistences which were identified and corrected. Common inconsistences were immediately communicated to field teams through their supervisors to ensure that timely actions are taken to correct the errors and to avoid future errors. Clean datasets were backed up at the end of each data collection day on a central computer and another copy on flash drive were kept with the survey data supervisor. After all data were collected and entered, they were transferred to STATA Version 14 for analysis [58]. Data were analyzed to get frequencies, percentages, proportions and means. Comparisons between groups, such as sub-counties or age strata was done using Chi Square or 2 × X tables. Indicators on OCV coverage were computed – first among all eligible household members, those who received only one dose of OCV, those who received two doses of OCV, and those who received at least one (one or two) doses of OCV. The dropout rate in OCV coverage was computed as household members who received the first dose but not the second dose of the vaccine.

Logistic regression was used to measure associations between OCV coverage with a set of respondents’ characteristics (predictors/factors) by generating odds ratios which were used to describe the likelihood of occurrence of an outcome in the exposed group in comparison to their unexposed counterparts. Various factors including district of residence, sex, education level, and knowledge about the vaccination campaign were included in the model. Initially, all variables were cross-tabulated with uptake of at least one dose of OCV and those that yielded a significance level below 20% (*p*-value < 0.2) were included in the multivariable regression using forward stepwise regression until the stopping criteria was met. The map was created using Arcmap software, version 10.5 [54]. The shapefiles used to create the maps were obtained from open-source portal of United Nations High Commission for the Refugees [59]. Geographical position system coordinates of the households were collected for all the households sampled. These coordinates were stored and used to locate the households on the maps.

### Quality assurance

To ensure quality, pretesting of study tools, training of RAs, field supervision of the RAs by the Principal investigator and co-investigators, routine data cleaning, correction of errors and verification visits to interviewed households were carried out. In addition, daily debrief meetings were held with RAs to discuss incidents or events which occurred during data collection. As a way of data verification, the survey supervisors revisited (15 households, about 10%) of the households after they were visited by data collectors to verify if the households were visited and interviewed. During the verification visits, no data were collected and the visits were brief lasting for 20 – 30 min.

## Results

### Demographic characteristics of the respondents

A total of 900 households and respondents were randomly enrolled in the survey. Each of the six districts had 150 households or respondents. Overall, more than half (504, 56%) of the respondents were male. About two thirds (585, 65%) of respondents were aged 20 – 49 years with over a third (306, 34%) being 50 years and above. Slightly more than half (477, 53%) of the respondents attained primary level of education, over a quarter 234, 26% achieved secondary level while 117, 13% reported no formal education at all.

### Oral cholera and COVID-19 vaccine coverages in the study districts

#### Oral cholera vaccine coverage

There were 4,315 household members of whom 85% received dose one of the OCV. All districts registered dose one coverage of above 79%. Obongi district registered the highest first dose coverage of 90%. Second dose coverage among these household members was 67% indicating a dropout of more than 10% between dose one and dose two. The lowest second dose coverage was recorded in Ntoroko district of 55%. The OCV vaccine coverage in the study populations in shown in Table 1.

**Table 1:** Oral cholera vaccine coverage by district, sex, age-group and education level for the six study districts of Uganda

**Background characteristics**	**Received only one dose of OCV,** **% (95%CI)**	**% Received two doses of OCV** **% (95%CI)**	**% Received at least one dose of OCV** **n, % (95%CI)**
**Overall**	**791, 18.3% (17.2—19.5)**	**2891, 67.0% (65.6—68.4)**	**3682, 85.3% (84.2—86.4)**
**District**			
Busia	93, 15.1% (12.4—18.2)	394, 64.0% (60.0—67.8)	487, 79.1% (75.6—82.2)
Kasese	155, 20.7% (17.8—23.7)	483, 64.3% (60.8—67.7)	638, 85.0% (82.2—87.4)
Madi-Okollo	79, 11.3% (9.0—13.9)	539, 77.0% (73.7—80.1)	618, 88.3% (85.7—90.6)
Namayingo	173, 25.2% (22.0—28.6)	424, 61.7% (58.0—65.4)	597, 86.9% (84.1—89.3)
Ntoroko	170, 25.1% (21.9—28.6)	375, 55.4% (51.6—59.2)	545, 80.5% (77.3—83.4)
Obongi	121, 13.7% (11.5—16.1)	676, 76.5% (73.5—79.2)	797, 90.2% (88.0—92.0)
**Sex**			
Male	382, 18.9% (17.2—20.7)	1332, 65.8% (63.7—67.9)	1714, 4.7% (83.0—86.2)
Female	409, 17.9% (16.3—19.5)	1559, 68.0% (66.1—69.9)	1968, 85.9% (84.4—87.3)
**Age group (years)**			
0–4	84, 15.0% (12.2—18.3)	290, 51.9% (47.6—56.1)	374, 66.9% (62.8—70.8)
5–9	102, 16.2% (13.4—19.3)	472, 73.4% (69.8—76.8)	576, 89.6% (87.0—91.8)
10–19	171, 15.6% (13.5—17.9)	816, 74.5% (71.8—77.1)	987, 90.1% (88.2—91.8)
20–29	144, 21.7% (18.6—25.0)	419, 63.0% (59.2—66.7)	563, 84.7% (81.7—87.3)
30–39	92, 18.4% (15.0—22.0)	335, 66.7% (62.4—70.8)	427, 85.1% (81.6—88.1)
40–49	90, 23.7% (19.5—23.3)	249, 65.5% (60.5—70.3)	339, 89.2% (85.6—92.1)
50 +	106, 22.5% (18.8—26.5)	310, 65.8% (61.3—70.1)	416, 88.3% (85.1—91.1)
**Education**			
None	95, 17.4% (14.3—20.8)	364, 65.7% (61.6—69.7)	459, 83.1% (79.8—86.2)
Primary	438, 18.6% (17.0—20.2)	1625, 68.8% (66.9—70.7)	2063, 87.4% (86.0—88.7)
Secondary	179, 16.2% (14.1—18.5)	741, 67.2% (64.3—69.9)	920, 83.4% (81.1—85.6)
Post-Secondary	79, 26.5% (21.0—32.9)	161, 53.8% (47.0—60.3)	240, 80.3% (74.6—85.3)

### Level of awareness about the OCV campaign and knowledge on the benefits of OCV

The vast majority (92%) of household heads had ever heard about OCV and 85% had heard about the planned OCV campaigns prior to the vaccination team visiting their homes. The percentage awareness by district were high: Namayingo (97%), Kasese (96%), Ntoroko (95%), Busia (91%), Obongi (87%) and Madi-Okollo (83%). The respondents also were aware of the benefits of OCV and the majority (88%) mentioned that OCV helps to prevent cholera infection, 13% mentioned that it prevents cholera outbreaks while another 5% mentioned that it prevents other diarrheal diseases.

### Barriers to uptake of oral cholera vaccine

**Fig. 2:**
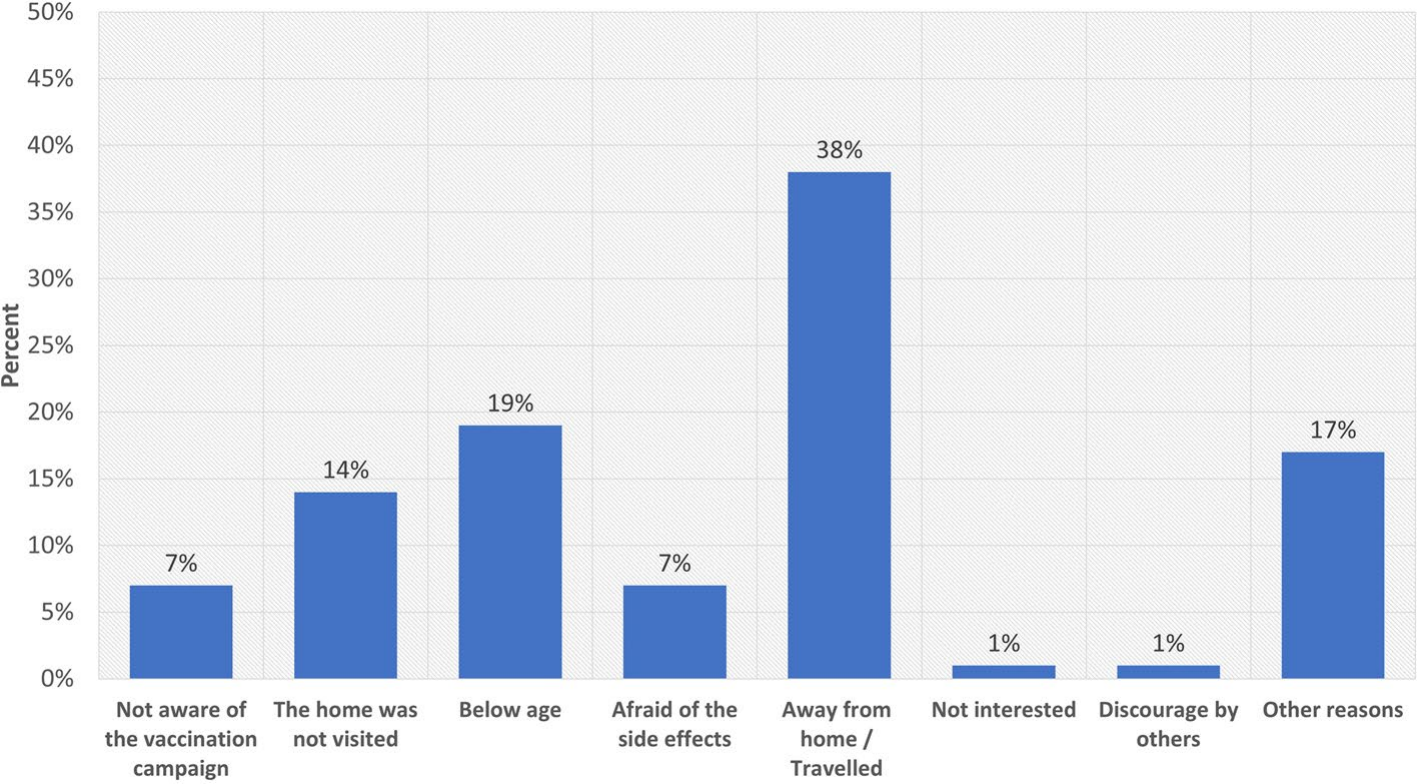
Reasons for missing oral cholera vaccination in the six study districts of Uganda

Among the 634 persons who did not get the OCV, the most common reasons given for missing out were: the individuals had travelled away from home (38%), below age (19%) and team did not visit the household (14%). The reasons given for missing out the OCV are shown in the Fig. 2.

### Determinants of uptake of the oral cholera vaccine

Oral cholera vaccine uptake was statistically significantly associated with education level and prior awareness about the vaccination campaign. Respondents with prior awareness were 3 times more likely [OR: 3.273, CI: 2.65–4.043] to report uptake of the OCV compared to their counterparts without prior information about the vaccination campaign. In addition, living in Madi-Okollo, Namayingo and Obongi districts was positively associated with OCV uptake. Determinants of OCV uptake are shown in Table 2.

**Table 2:** Determinants of uptake of at least one dose of OCV in the six study districts of Uganda

**Characteristic**	**Un adjusted odds ratio (95% CI)**	**Adjusted odds ratio (95% CI)**
**District**		
Busia	Ref	Ref
Kasese	1.496 (1.132–1.976) *	1.299 (0.966–1.747)
Madi-Okollo	1.996 (1.477–2.698) ***	1.894 (1.373–2.613) ***
Namayingo	1.757 (1.309–2.359) ***	1.531 (1.115–2.104) **
Ntoroko	1.094 (0.833–1.435)	0.981 (0.731–1.317)
Obongi	2.427 (1.808–3.257) ***	2.116 (1.545–2.899) ***
**Sex**		
Male	Ref	Ref
Female	1.095 (0.925–1.296)	1.113 (0.931–1.33)
**Age group (years)**		
0–4	Ref	Ref
5–9	4.253 (3.124–5.788) ***	4.344 (3.164–5.962) ***
10–19	4.521 (3.466–5.895) ***	4.568 (3.473–6.010) ***
20–29	2.730 (2.074–3.594) ***	3.024 (2.271–4.026) ***
30–39	2.816 (2.082–3.809) ***	2.936 (2.148–4.014) ***
40–49	4.090 (2.828–5.914) ***	3.052 (3.052–6.515) ***
50 +	3.741 (2.684–5.214) ***	4.269 (3.021–6.033) ***
**Education**		
No formal education	Ref	Ref
Primary	1.403 (1.088–1.808) **	1.539 (1.177–2.014) **
Secondary	1.019 (0.775–1.339)	1.182 (0.878–1.591)
Post Sec	0.824 (0.574–1.183)	1.168 (0.785–1.739)
**Had heard about the vaccination campaign prior to the vaccination team visiting their home**		
No	Ref	Ref
Yes	3.256 (2.664–3.980) ***	3.273 (2.65–4.043) ***

* *p* < 0.05, ** *p* < 0.01, ***p < 0.001

#### COVID-19 vaccine coverage in the six study districts of Uganda

There were 2,085 household members aged 18 years and above, 70% had received at least one dose of the COVID-19 vaccine. Kasese district with 95% COVID-19 dose one vaccine coverage was the highest and the lowest was in Namayingo district at 33.2%. The COVID-19 vaccine coverage by the background characteristics are shown in Table 3.

**Table 3:** Percentage of household members aged > 18 years that received COVID-19 vaccine in the six study districts of Uganda

**Background characteristics**	**% Received dose 1 of Covid-19 vaccine**	**% Received dose 2 of Covid-19 vaccine**		**Number of households member (n)**
		**Yes**	**N/A**	
**Overall**	69.8 (67.8–71.8)	18.8 (17.1–20.5)	21.4 (19.6–23.2)	**2085**
**District**				
Busia	38.2 (33.0–43.6)	21.6 (17.3–26.4)	0.3 (0.0–1.6)	338
Kasese	94.9 (92.0–96.9)	22.9 (18.6–27.6)	28.9 (24.2–33.9)	350
Madi-Okollo	88.7 (84.7–91.9)	19.8 (15.6–24.6)	46.2 (40.6–51.9)	318
Namayingo	33.2 (28.3–38.5)	12.1 (8.9–16.1)	0.0	346
Ntoroko	77.7 (72.8–82.1)	23.2 (18.8–28.1)	7.8 (5.2–11.3)	332
Obongi	84.8 (80.9–88.2)	14.2 (10.9–18.0)	42.9 (38.0–47.9)	401
**Sex**				
Male	71.0 (67.9–73.9)	22.8 (20.1–25.6)	19.7 (17.2–22.4)	909
Female	69.0 (66.2–71.6)	15.7 (13.7–17.9)	22.8 (20.4–25.3)	1176
**Age group (year)**				
18–19	67.2 (54.6–78.2)	11.9 (5.3–22.1)	29.9 (19.3–42.3)	67
20–29	67.7 (64.0–71.2)	13.2 (10.8–16.0)	24.8 (21.6–28.3)	665
30–39	66.3 (62.0–70.5)	18.5 (15.2–22.2)	18.9 (15.6–22.6)	502
40–49	76.6 (72.0–80.7)	24.7 (20.5–29.4)	20.3 (16.3–24.7)	380
50 +	71.5 (67.2–75.6)	23.1 (19.4–27.2)	19.1 (15.7–23.0)	471
**Education**				
No formal education	72.5 (66.5–77.9)	15.1 (10.9–20.2)	25.1 (19.9–30.9)	255
Primary	69.1 (66.3–71.8)	15.2 (13.1–17.4)	23.5 (21.1–26.1)	1118
Secondary	68.1 (64.0–72.0)	23.4 (19.9–27.1)	16.4 (13.4–19.8)	548
Post-Secondary	76.2 (69.0–82.5)	32.3 (25.2–40.1)	18.3 (12.7–25.1)	164

### Level of awareness about the COVID-19 vaccination, treatment and benefits

A high percentage (97%) had ever heard about the COVID-19 vaccines and 89% also knew a place where treatment for COVID-19 patients was provided. This knowledge was high in all districts with Kasese (97%), Busia (97%), Ntoroko (90%), Madi-Okollo (86%), Obongi (83%) and Namayingo (78%). The majority, 83% of respondents knew the benefits of the COVID-19 vaccine and mentioned that it helps to prevent COVID-19 infection, 24% mentioned that it prevents severe infection while less than 1% mentioned that it had no benefit. The main sources of information about COVID-19 were the radios (59%), local leaders (44%) and health workers (42%) as shown in Additional file 2.**.**

### Barriers to uptake of COVID-19 vaccines in the six study districts of Uganda

**Fig. 3:**
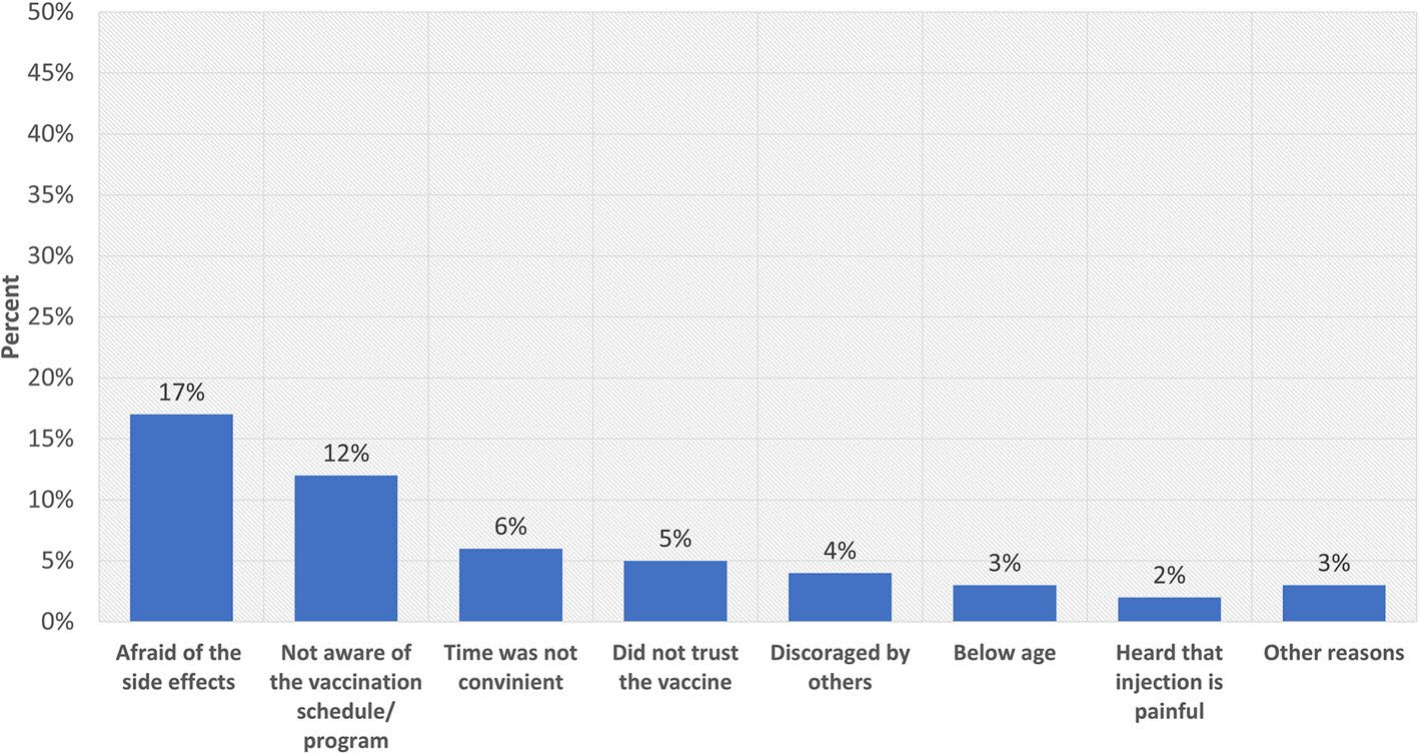
Reasons for missing COVID-19 vaccination in the six study districts of Uganda

The respondents were asked to give the reasons why they could not receive the vaccination. The most common response in 17% of the respondents who missed was that they were afraid of the vaccine side effects. The reasons for missing COVID-19 vaccination are shown in Fig. 3.

#### Distance to the nearest facility

The study found that majority (69%) of respondents travelled on foot to the nearest health facility to get the services, 25% used a bicycle or motorcycle, 3% used boats and another 3% used a taxi or bus.

### Vaccine adverse events following immunizations (AEFI)

#### Oral cholera vaccine

**Fig. 4:**
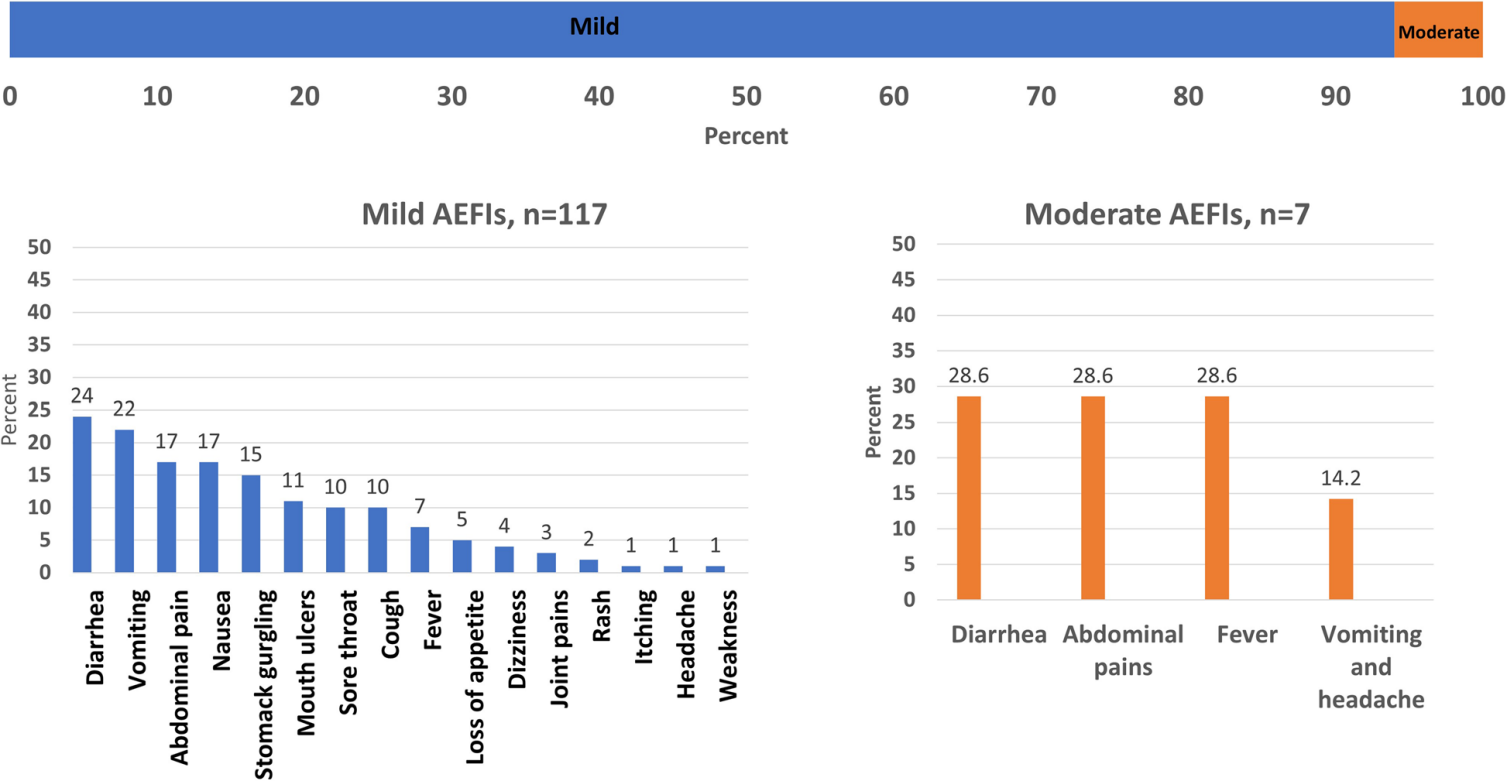
Reported adverse events following immunization with COVID-19 vaccines in the six study districts of Uganda

The percentage of persons in households who were reported to have had AEFI as a result of receiving OCV was small, 2%, 123/4,315. Among the 123 household members who reported AEFI, the vast majority (117/123, 94%) rated the AEFI as mild, while (6%, 7/123) of the cases were moderate. There were no severe AEFI reported. The AEFI reported during the study are shown in Fig. 4.

#### COVID-19 vaccines

**Fig. 5:**
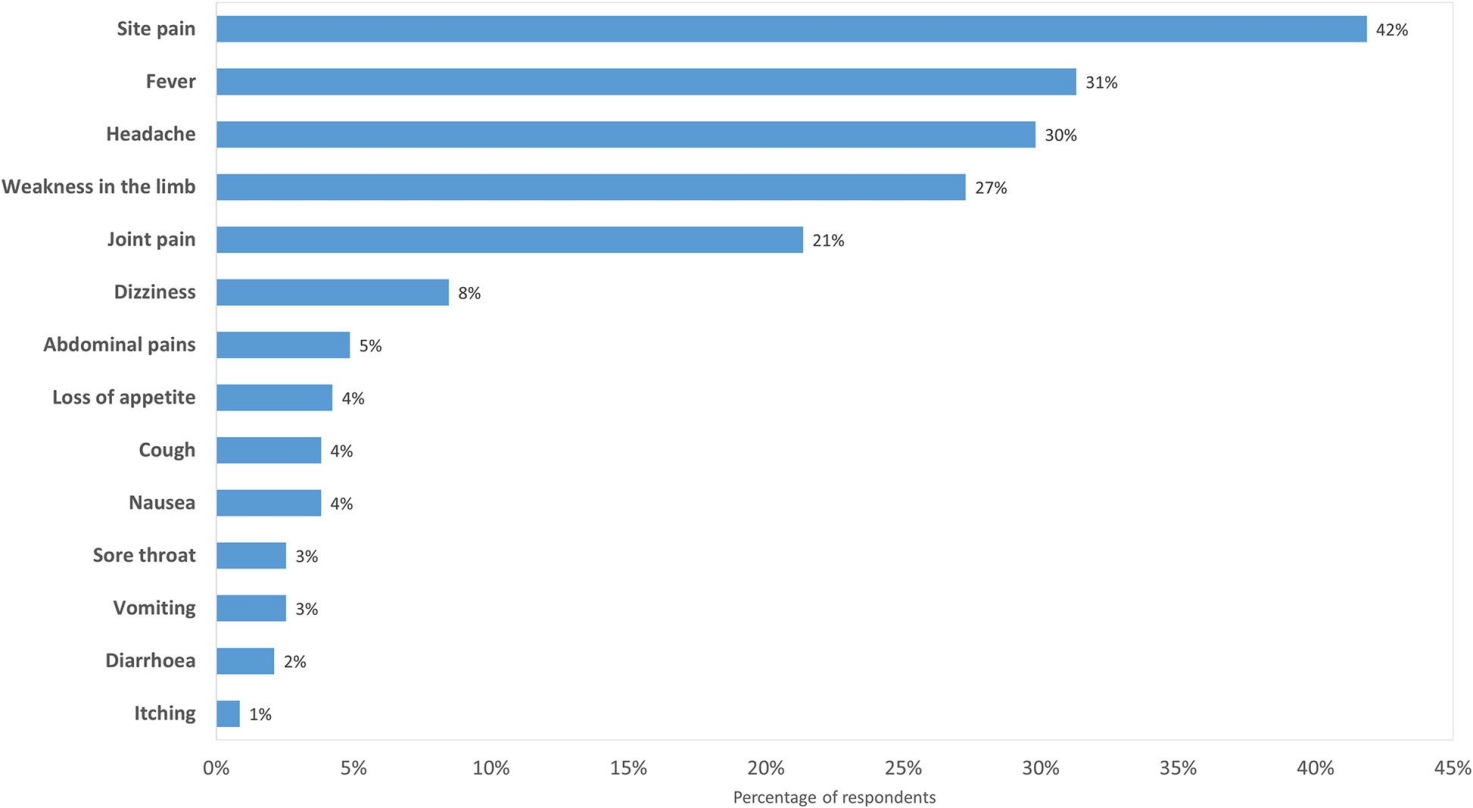
Reported AEFI after COVID-19 vaccination in the six study districts of Uganda

Among the 2,085 respondents who were above 18 years and received the COVID-19, 473 (22.69%) reported getting AEFI. Pain at the site of injection was the commonest AEFI reported in 42% of persons. Other AEFI are shown in Fig. 5.

Among the 478 respondents who reported AEFI with the COVID-19 vaccine, the vast majority (94%) rated the AEFI as mild (resolved, a person continued with normal work), 6% (28 persons) of the cases had moderate (interfered with work but resolved without seeking care) while only one person was severe (sought care from the nearest health workers) and presented with the following severe symptoms namely: fever, vomiting and cough.

### Water, sanitation and hygiene coverages in the six study districts of Uganda

**Fig. 6:**
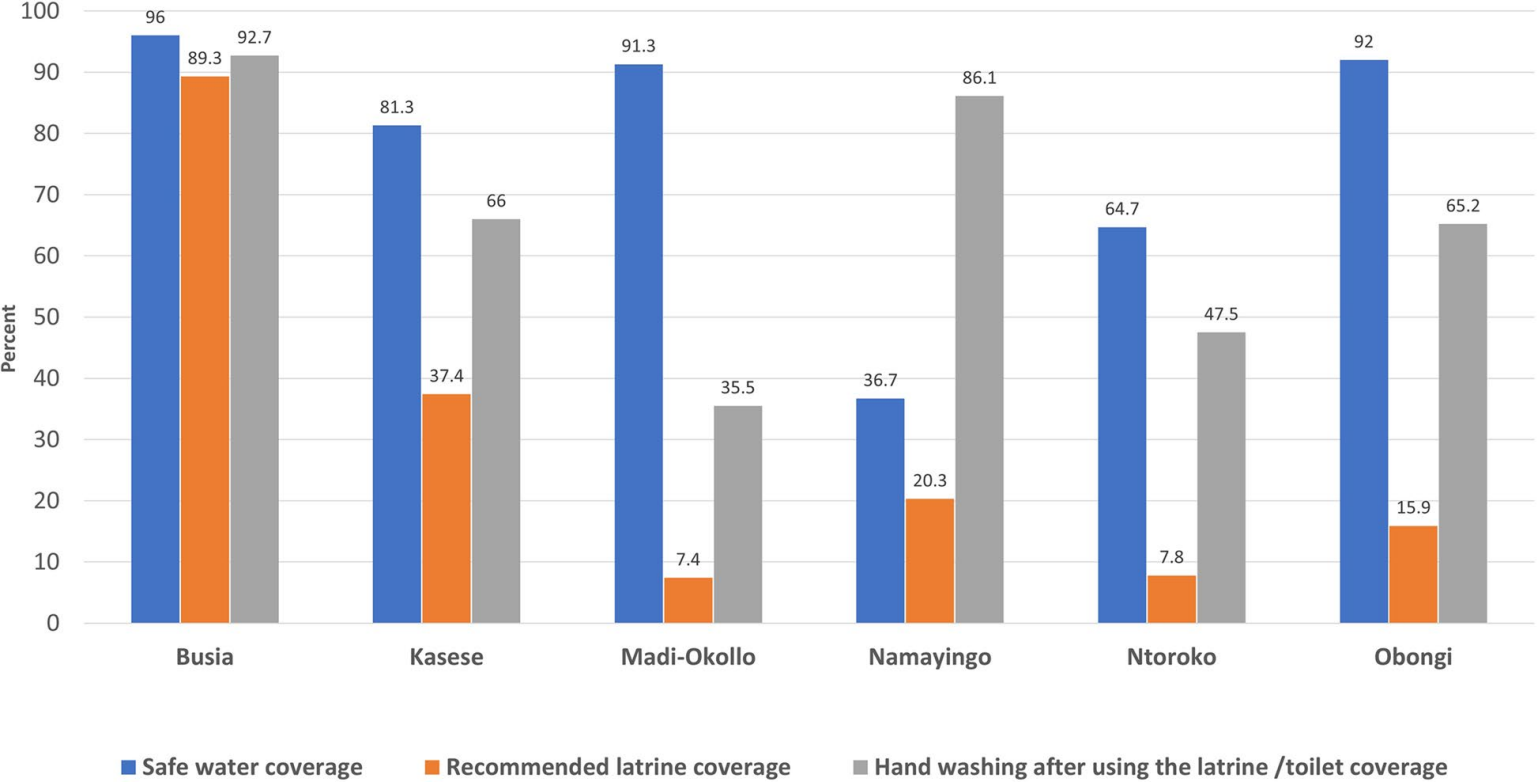
Status of WASH in the six study districts of Uganda

With exception of Busia districts where the WASH condition coverages were above 89%, in the rest of the districts the latrine coverages were below 40% with the lowest of 7.4% in Madi-Okollo district. Hand-washing practice in these districts were also low and ranged from 35.5% in Madi-Okollo to 66% in Kasese. The status of WASH conditions in the six districts is shown in Fig. 6.

#### Safe water coverage

The study showed that three quarters (75%) of households used the recommended protected water sources. However, 17% of households used surface water sources. In Namayingo district, the majority, 63% of the households used surface water or unprotected water sources. The main water sources used by the households in the six districts are shown in the Table 4.

**Table 4:** The main source of water used by the households in the six study districts of Uganda

**Background characteristics**	**Public taps or stand pipes,** **% (95%CI)**	**Boreholes or tube wells,** **% (95%CI)**	**Protected spring,** **% (95%CI)**	**Protected dug well,** **% (95%CI)**	**Surface source (dam, lake, river, stream, pond and canal),** **% (95%CI)**	**Unprotected dug well,** **% (95%CI)**	**Others,** **% (95%CI)**	**Number of households (n)**
**Overall**	**39.1 (35.9—42.4)**	**32.9 (29.8—36.1)**	**2.7 (1.7—3.9)**	**2.3 (1.5—3.5)**	**16.8 (14.4—19.4)**	**3.4 (2.4—4.9)**	**2.8 (1.8—4.1)**	**900**
**District**								
Busia	61.3 (53.0—69.2)	19.3 (13.3—26.6)	12.7 (7.8—19.1)	2.7 (0.1—6.7)	0	0.7 (0.02—3.7)	3.3 (1.1—7.6)	150
Kasese	71.3 (63.4—78.4)	6.7 (3.2—11.9)	0	3.3 (1.1—7.6)	16 (10.5—22.7)	0.7 (0.02—3.7)	2 (0.4—5.7)	150
Madi-Okollo	28 (21.0—35.9)	59.3 (51.0—67.3)	3.3 (1.1—7.6)	0.7 (0.02—3.7)	6.0 (2.8—11.1)	2 (0.4—5.7)	0.7 (0.02—3.7)	150
Namayingo	8.7 (4.7—14.4)	27.3 (20.4—35.2)	0	0.7 (0.02—3.7)	48 (39.8—56.3)	8 (4.2—13.6)	7.3 (3.7—12.7)	150
Ntoroko	38.0 (30.2—46.3)	20 (13.9—27.3)	0	6.7 (3.2—11.9)	26 (19.2—33.8)	9.3 (5.2—15.2)	0	150
Obongi	27.3 (20.4—35.2)	64.7 (56.5—72.3)	0	0	4.7 (1.9—9.4)	0	3.3 (1.1—7.6)	150
**Education**								
No formal education	35.6 (27.3—45.1)	36.4 (28.1—45.9)	2.5 (0.5—7.1)	2.5 (0.5—7.1)	16.1 (9.8—23.6)	4.2 (1.4—9.5)	2.5 (0.5—7.1)	120
Primary	34.2 (30.0—38.7)	36.8 (32.4—41.3)	2.7 (1.5—4.7)	2.5 (1.3—4.4)	17.8 (14.4—21.5)	3.2 (1.8—5.2)	2.7 (1.5—4.7)	473
Secondary	44.7 (38.3—51.3)	26.6 (21.1—32.7)	2.5 (0.9—5.4)	1.7 (0.5—4.3)	17.3 (12.7—22.7)	3.8 (1.8—7.1)	3.4 (1.5—6.5)	237
Post-Secondary	58.6 (46.2—70.2)	21.4 (12.5 -32.9)	2.9 (0.3—9.9)	2.9 (0.3—9.9)	10 (4.1—19.5)	2.9 (0.3—9.9)	1.4 (0.0—7.7)	70

### Boiling or treating of drinking water

Boiling of drinking water in the districts was below 50% except in Busia (55.3%). Majority of the households (61%) mentioned that they neither boil nor treat drinking water. The findings on boiling or treatment of drinking water are shown in Additional file 3.

### Availability of sanitary facilities (latrines and toilets)

Majority of households (68%) did not have the recommended sanitary facilities (latrines or toilets). More than a third (37%) of the households had no sanitary facilities. The households that had sanitary facilities are shown in Table 5.

**Table 5:** Percent of households that had a latrine / toilet facility by district and by education level of the household heads in the six study districts of Uganda

**Background characteristics**	**Has no access to recommended toilet / latrine type (%)**	**Has access to recommended toilet / latrine type (%)**	**Ownership of recommended toilet / latrine type**					**Number of households (n)**
			**Traditional Pit Latrine (with a washable floor)**	**VIP Latrine**	**Ecological sanitation toilet**	**Pour Flush**	**Flush Toilet/WC**	
**Overall**	**68.5 (65.4–71.6)**	**31.5 (28.5–34.7)**	**23.1 (20.4–26.0)**	**4.7 (3.4–6.3)**	**1.6 (0.9–2.6)**	**1.4 (0.8–2.5)**	**0.7 (0.2–1.4)**	**900**
**District**								
Busia	10.7 (6.2–16.7)	89.3 (83.3–93.8)	65.3 (57.1–72.9)	18.0 (12.2–25.1)	0.7 (0.0–3.7)	2.0 (0.4–5.7)	3.3 (1.1–7.6)	150
Kasese	62.6 (54.4–70.4)	37.4 (29.6–45.6)	28.7 (21.6–36.6)	5.3 (2.3–10.2)	0.7 (0.0–3.7)	2.0 (0.4–5.7)	0.7 (0.0–3.7)	150
Madi-Okollo	92.6 (87.3–96.3)	7.4 (3.7–12.7)	2.5 (0.7–6.7)	0.8 (0.0–3.7)	4.1 (1.5–8.5)	0.0	0.0	150
Namayingo	79.7 (72.7–86.1)	20.3 (13.9–27.3)	19.4 (13.3–26.6)	0.9 (0.0–3.7)	0.0	0.0	0.0	150
Ntoroko	92.2 (86.4–95.8)	7.8 (4.2–13.6)	5.0 (2.3–10.2)	0.0	0.0	2.8 (0.7–6.7)	0.0	150
Obongi	84.1 (77.1–89.5)	15.9 (10.5–22.7)	9.8 (5.7–16.0)	0.8 (0.0–3.7)	4.5 (1.9–9.4)	0.8 (0.0–3.7)	0.0	150
**Education**								
No formal education	85.4 (77.9–91.4)	14.6 (8.6–22.1)	8.7 (4.1–15.0)	4.9 (1.9–10,7)	1.0 (0.0–4.6)	0.0	0.0	118
Primary	73.6 (69.4–77.5)	26.4 (22.5–30.6)	19.7 (16.2–23.5)	2.5 (1.3–4.4)	2.2 (1.0–3.9)	1.5 (0.6–3.0)	0.5 (0.0–1.5)	473
Secondary	61.6 (55.1–67.8)	38.4 (32.2–44.9)	29.5 (23.8–35.8)	5.8 (3.3–9.7)	0.9 (0.1–3.0)	1.3 (0.3–3.7)	0.9 (0.1–3.0)	237
Post-Secondary	32.8 (22.1–45.1)	67.2 (54.9–77.9)	44.8 (32.4–56.7)	14.9 (7.1–24.7)	1.5 (0.0–7.7)	3.0 (0.3–9.9)	3.0 (0.3–9.9)	70

There were variations in availability of latrines with education level of the respondents. The higher the education the more likely the respondents were to have the recommended latrines. These differences were statistically significant at each higher education level

### Hand-washing after visiting a toilet

Among 802 households that owned sanitary facilities (latrines or toilets), two thirds (66%) reported that they always washed hands after using a toilet while 3% never wash hands after using a toilet or latrine. The hand-washing practices by districts are shown in Table 6.

**Table 6:** Percent of respondents that washed hands after visiting the toilet/latrine in the six study districts of Uganda

Background characteristics	Always (%)	Sometimes (%)	Rarely (%)	Never (%)	Number of households (n)
**Overall**	**65.7 (62.3–69.0)**	**23.3 (20.4–26.4)**	**8.0 (6.2–10.1)**	**3.0 (1.9–4.4)**	**802**
**District**					
Busia	92.7 (87.3–96.3)	7.3 (3.7–12.7)	0.0	0.0	150
Kasese	66.0 (57.8–73.5)	26.0 (19.2–33.8)	6.0 (2.8–11.1)	2.0 (0.4–5.7)	150
Madi-Okollo	35.5 (27.0–44.8)	33.9 (25.5–43.0)	23.1 (16.0–31.7)	7.4 (3.5–13.7)	121
Namayingo	86.1 (78.1–92.0)	13.9 (8.0–21.9)	0.0	0.0	108
Ntoroko	47.5 (39.1–56.1)	37.6 (29.6–46.1)	7.8 (4.0–13.5)	7.1 (3.4–12.7)	141
Obongi	65.2 (56.4–73.2)	21.2 (14.6–21.2)	12.1 (7.1–18.9)	1.5 (0.18–5.4)	132
**Education**					
No formal education	63.1 (53.4–72.7)	22.3 (14.6–31.3)	8.7 (4.0–15.8)	5.8 (2.1–12.1)	104
Primary	61.4 (56.5–66.2)	24.8 (20.7–29.3)	10.6 (7.8–14.0)	3.2 (1.7–5.4)	407
Secondary	71.9 (65.5–77.7)	21.4 (16.2–27.4)	4.9 (2.5–8.6)	1.8 (0.5–4.5)	224
Post-Secondary	76.1 (64.1–85.7)	20.9 (11.9–32.6)	1.5 (0.0–8.0)	1.5 (0.0–8.0)	67

There were differences in hand-washing practices between districts and with the education levels of the respondents. However, these differences between the education levels were not statistically significant

## Discussion

This study generated comprehensive data for prevention of both cholera and COVID-19 pandemics in the six hotspot districts of Uganda. Most importantly, the data on vaccine coverage, AEFI and WASH conditions could be used to guide future preventive interventions of the two pandemics in affected communities of Uganda. In regards to cholera prevention, the study found high vaccination coverage for OCV for both the first and the second dose campaign in all the six study districts. For the first OCV dose, all districts achieved the coverage of above 79%. The second dose average coverage was lower than the first dose at 67%. Ntoroko district recorded the least second dose coverage of 55% which was still higher than the 50% that is required for the community to acquire herd immunity against cholera [38]. The high vaccination coverage registered in these districts following OCV campaigns and the subsequently acquired herd immunity could be responsible for the reported zero cholera outbreaks in these districts of Uganda during the period that followed the vaccination campaigns up to the end of 2021 as established in another study on cholera in Uganda [60].

The vaccination coverage for COVID-19 vaccines in the same communities were however, lower than for OCV coverage. The second dose of COVID-19 vaccine coverage for the targeted population (persons aged 18 years and above) were at an average of 18% for the six study districts. This COVID-19 average coverage was far below the United Nations recommended level of 70% vaccination coverage of the target group [39]. The very low COVID-19 vaccination coverage could be attributed to a number of factors.

First, the mass vaccination campaign strategy employed to administer COVID-19 vaccines that was fixed posts (either at health facility level or outreach post) created accessibility challenges as opposed to the mobile *house-to-house mass vaccination strategy* that was used for OCV. In addition, since most of the individuals in the study area walked on foot to reach the fixed points, it is possible that the elderly, persons with walking disabilities and are weak, and those with other urgent commitments and businesses opted-out. In this case, to scale-up COVID-19 vaccine uptake, use of *a mobile strategy* could be explored. This is necessary since increasing the COVID-19 vaccine coverage is important to protect the communities against the evolving COVID-19 global threat [61]. However, there is financial implication for adapting *mobile strategy* for COVID-19 mass vaccination campaigns. It should be noted that cholera pandemic in Uganda occur in particular locations, hotspot districts and communities [42, 62] and not the whole country as is the case with COVID-19 [63]. Therefore, for COVID-19 mass vaccination to be conducted by use of *mobile vaccination strategy*, additional operational funds are needed. Getting these extra funds may not be easy following the effects of COVID-19 on the economies [64]. Hence other alternative cost-effective approaches have to be considered including integration of COVID-19 vaccines into the already existing routine immunization strategy.

Secondly, the commonest reason given by the respondents who missed COVID-19 vaccination was fear of the vaccine side effects. Indeed, many respondents in this study reported moderate and some severe AEFI after COVID-19 vaccines. Similarly, studies in other countries have documented AEFI with COVID-19 vaccines [48, 65, 66]. Consequently, this could have resulted in COVID-19 vaccine hesitancy as previously documented elsewhere in Africa [19, 67, 68]. Hence, further specific studies are required to get better understanding of the COVID-19 vaccines hesitancy. Interestingly, there were no severe AEFI reported among the OCV recipients in all the study districts. This findings are in agreement with that of other study which also reported no severe AEFI following mass OCV campaigns [69].

Thirdly, unavailability of COVID-19 vaccines at the health posts could be a limiting factor leading to low coverage since it is a well-documented fact that countries in Africa had difficulties in acquiring the needed vaccines due to inequity in COVID-19 vaccine access as a consequence of the vaccine manufacturers prioritizing their own countries and people in the developed nations [70]. We think that Uganda is not unique and was affected as well. This school of thought is also supported by another study on factors associated with COVID-19 vaccine hesitancy in Uganda which showed that Uganda needed a total of 45 million doses of COVID-19 vaccines (if all vaccines provided were of two doses) to vaccinate eligible population of 22 million people (18 years and above) in a phased manner yet by March 2021, the government had received only 964,000 doses [71]. Therefore, ensuring availability of adequate COVID-19 vaccines at the fixed posts, in Uganda and Africa at large is important for COVID-19 prevention. In this regard, steps by the Africa Centers for Disease Control (CDC) to promote COVID-19 vaccine production / manufacture of vaccines in Africa [72] should be supported. Meanwhile, there is a need for the implementers in Uganda to incorporate trust building measures in community mobilization and engagements to attract more people to be vaccinated. In addition, in order to get deeper understanding of the factors responsible for the low uptake of COVID-19 vaccines, further qualitative studies that involve interviews with key informants and groups in the communities are recommended.

The sustained promotion of safe WASH and waste management practices in the communities and in the public places serve as important interventions to control transmission and spread of several infectious disease such as the COVID-19 and cholera [73]. However, often after OCV campaigns, vaccine coverage surveys are done [40, 53] but they omit assessing for WASH status which if adequate is an important pillar for healthy living [50]. The WASH condition data generated in this study could be used address the immediate WASH gaps and to strengthen long term cholera preventive planning. Previously, there were attempts to collect WASH data during coverage surveys [74]. However, the data collected were not adequate for all WASH conditions. Furthermore, the study revealed suboptimal WASH coverages in some districts. For example, the latrine and/or toilet coverages in the five districts of Obongi, Madio-Okollo, Namayingo and Kasese were between 7.4%—37.4% implying that a significant percentage (62.6%—92.6%) of the populations in these districts had no access to the recommended sanitary facilities and were practicing open defecation. Therefore, to consolidate the benefits of OCV and to ensure access to safe WASH conditions in line with Sustainable Development Goal (SDG 6) [75], special efforts are needed to promote WASH conditions in the five study districts.

Additionally, since the protection with OCV is for about three to five years [76] and improvement in WASH conditions takes a long time than the protective period for OCV, there is a need for the future follow up vaccination campaigns to consolidate the preventive achievements. Hence, this study could be a suitable reference document to support future OCV applications from Uganda to Global Task Force for Cholera Control. Besides, this study findings on WASH conditions in communities could be used by stakeholder to assess progress toward ensuring universal access to safe water and sanitation by 2030 as per United Nations’ Sustainable Development Goal 6 [75].

### Strength and limitations of this study

To our knowledge, this is the first study to comprehensively incorporate WASH assessment into OCV and COVID-19 vaccine coverage survey. This is important since the data generated can be used by the actors to simultaneously plan and prevent the two major ongoing pandemics in Uganda and the world [10]. Despite these strengths, there were also a number of limitations. First, calculation of sample size in a study involving two vaccine campaigns with different target age groups. The target age for OCV vaccination was one year and above while that of COVID-19 vaccine coverage was 18 years or more. Second, estimation of the number of households to enroll in the study given that the target age group is not the same for each type of vaccines 1 years and above for OCV and 18 years and above for COVID-19 vaccines. Third, recall bias for both OCV and COVID-19 vaccination since data collection was done after two months.

There were also some study findings that were not fully understood and explained namely, the odds of receiving OCV though high for both secondary and post-secondary levels than with no education reduced with increasing education levels. We could not explain this reduction in odds ratio with education levels. Hence, further studies are required to provide more information. Similarly, although the coverage for OCV were high for both doses (one and two), we could not explain the high OCV second dose dropout rate of more than 10% between the doses since in both instances (first and second dose administration) house-to–house vaccine delivery strategy which ensure finding the recipient at their residence. Thus, additional studies are needed to identify factors that could have resulted in this high dropout rate.

Also, we could not fully explain why Busia district was a cholera hotspot yet it had good WASH coverage. It is a known fact that availability of WASH infrastructure and use are two different things [77]. Furthermore, being a border district it is possible that cross-border activities are at play for frequent cholera outbreaks as documented elsewhere [78]. However, there is no specific literature to support these postulations. Hence, more studies are needed to provide information on utilization of WASH services.

## Conclusion

This study highlights the importance of mass vaccination campaigns in preventing cholera and COVID-19 pandemics. While vaccine coverage for OCV was high and above the recommended protective level, vaccine hesitancy by the community led to low coverage for COVID-19 vaccination. The study also showed that integrating WASH condition assessment tools in cholera vaccine coverage surveys is feasible and can generate more data that could be used for addressing immediate gaps for the long-term cholera prevention and for monitoring progress toward achieving some SDGs [75]. Further studies are recommended to provide more information about COVID-19 vaccine hesitancy.

## Supplementary Information

**Additional file 1.** WASH assessment tool.

**Additional file 2.** The sources of information on COVID-19.

**Additional file 3.** Percent of households that boiled or treated drinking water by district and educational level in the six study districts of Uganda.

## Data Availability

The datasets generated and/or analyzed during the current study are not publicly available due sensitivity of the medical information therein and but are available from the corresponding author on reasonable request after relevant ethical clearance.

## Abbreviations

AEFI: Adverse events following immunization
COVID-19: Coronavirus Disease, 2019
OCV: Oral cholera vaccine
SDG: Sustainable Development Goals
UNICEF: United Nations Children’s Emergency Fund
WASH: Water, sanitation and hygiene
WHO: World Health Organization
