# Supplementary material for: Cholera and COVID-19 pandemic prevention in multiple hotspot districts of Uganda: vaccine coverage, adverse events following immunization and WASH conditions survey
Source: BMC Infect Dis. 2023 Jul 21;23:487. doi: 10.1186/s12879-023-08462-y (PMC10362646; doi:10.1186/s12879-023-08462-y)
Supplement: Supplementary file 1 — Additional file 1. WASH assessment tool. [file 12879_2023_8462_MOESM1_ESM.pdf]

WATER, SANITATION AND HYGIENE ASSESSMENT TOOL

**Assessment Dates:**	
**Locations:**	
**Objective:**	
**Assessment team:**<br>**(names and positions)**	

SECTION A: HOUSEHOLD QUESTIONNAIRE

(should be administered to the household head)

A1	Availability of safe water	Response
1.	What is the main type of water source used by your household?	☐ Public taps or stand pipes<br>☐ Boreholes or tube wells<br>☐ Protected dug well<br>☐ Unprotected dug well<br>☐ Protected spring<br>☐ Unprotected spring<br>☐ Surface source (dam, lake, river, stream, pond and canal)<br>☐ Covered rainwater tank<br>☐ Uncovered rainwater tank<br>☐ Cart with tank/drum<br>☐ Other (specify)
2.	Are there times when you buy water from vendors?	☐ Yes<br>☐ No
3.	If yes, how much do you pay for the water from the vendors per 20 liter jerrycan?	______________Shs.
4.	How far (in Kms) is the main water source to your household?	______________Kms
5.	How long (hours to and from) does it take you to collect water from the main water source?	______________Hours
6.	How many 20-litre jerrycans of water do you use in this Household per day?	_________________
	Do you use water from other sources, other than what is brought ate home?	
7.	Is the water you use in your household for drinking boiled or treated?	Yes, boiled…………………………..1<br>Yes, treated………………………..2<br>Both boiled and treated…….3<br>Neither boiled nor treated…4

A2	Availability of safe sanitary facility	Response
8.	Does this household have a latrine/toilet facility?	☐ Yes<br>☐ No
9.	If yes, of what kind?	☐ Traditional Pit Latrine (with a washable floor)<br>☐ Traditional Pit Latrine (with a non-washable floor)<br>☐ Pit latrine with no slab (non-washable)<br>☐ VIP Latrine<br>☐ Pour Flush<br>☐ Flush Toilet/WC<br>☐ Ecological sanitation toilet<br>☐ Other (specify)<br>___________________________
10.	Is there a separation for males and females (communal setting)	☐ Yes<br>☐ No<br>☐ N/A
11.	Do you share this facility with others who are not members of your household? *(This also includes those using public toilets)*	☐ Yes<br>☐ No
12.	If you share, under what conditions? *(Do you pay to use the facility?)*	☐ Public facility/ communal<br>☐ Private-shared<br>☐ Private-shared<br>☐ Others (specify)
13.	About how many people share your facility? (*Ask the respondent for the approximate number of users)*	
14.	Do you pay to use the facility?	☐ Yes<br>☐ No
	**Hand washing practices**	
15.	After visiting a latrine/toilet, do you wash your hands?	☐ Always<br>☐ Sometimes<br>☐ Rarely<br>☐ Never
16.	If rarely or never, what prevents you from washing hands after using latrine/toilet?	☐ Shortage of water<br>☐ Shortage of soap<br>☐ No container<br>☐ No interest / not important<br>☐ Not aware<br>☐ Other (specify)<br>____________________________
17.	Before eating food, do you wash your hands?	☐ Always<br>☐ Sometimes<br>☐ Rarely<br>☐ Never

A2	Availability of safe sanitary facility	Response
18.	If rarely or never, what prevents you from washing hands before eating food?	☐ Shortage of water<br>☐ Shortage of soap<br>☐ No container<br>☐ No interest / not important<br>☐ Not aware<br>☐ Other (specify)<br>___________________________

Household GPS GIS coordinates. ---------------------------

SECTION B: HOUSEHOLD OBSERVATION CHECKLIST
(The investigator makes direct observation in the visited households)

	Aspect	Yes=1	No=2
**B1**	**Type of House**		
1.	Permanent (Brick with iron sheets / tiled roof)		
2.	Semi-permanent (Mud and Wattle with iron sheets		
3.	Temporary (mud and wattle, grass thatched)		
4.	Compound cleanness (clean, no rubbish littered, not bushy)		
5.	Other (specify)		
	**Household GPS GIS coordinates. Latitude ____________________Longitude________________**		

Section B2. Direct observation and inspection of the WASH facilities

No	(1) NAME OF HOUSEHOLD HEAD OR APPROPRIATE CODE	(2) LATRINE									(3) WATER CHAIN																(4) KITCHEN	
		(a) Avail-ability		(b) Type		(c) Con-dition		(d) Hand Washing Facility			(a) Water Source										(b) Storage container (s)		(c) Method of treatment of drinking Water				(a) Drying Rack	
								Present			(i) Protected					(ii) Not protected												
		Present	Not present	Un-improved	Improved (Washable floor)	Clean	Dirty	With soap/ ash	Without soap/ash	Not present	Bore hole	Tap water	Shallow well	Protected spring	Rain water tank	Lake	River/ Stream	Unprotected spring	Broken down well	Water hole	Narrow mouthed	Wide mouthed	Boiled	Chlorinated	Filtered	Not treated	Present	Not present
1																												
2																												
3																												
4																												
5																												
6																												
7																												
8																												
9																												
10																												
