# Supplementary figures and images for: Cholera and COVID-19 pandemic prevention in multiple hotspot districts of Uganda: vaccine coverage, adverse events following immunization and WASH conditions survey
Source: BMC Infect Dis. 2023 Jul 21;23:487. doi: 10.1186/s12879-023-08462-y (PMC10362646; doi:10.1186/s12879-023-08462-y)

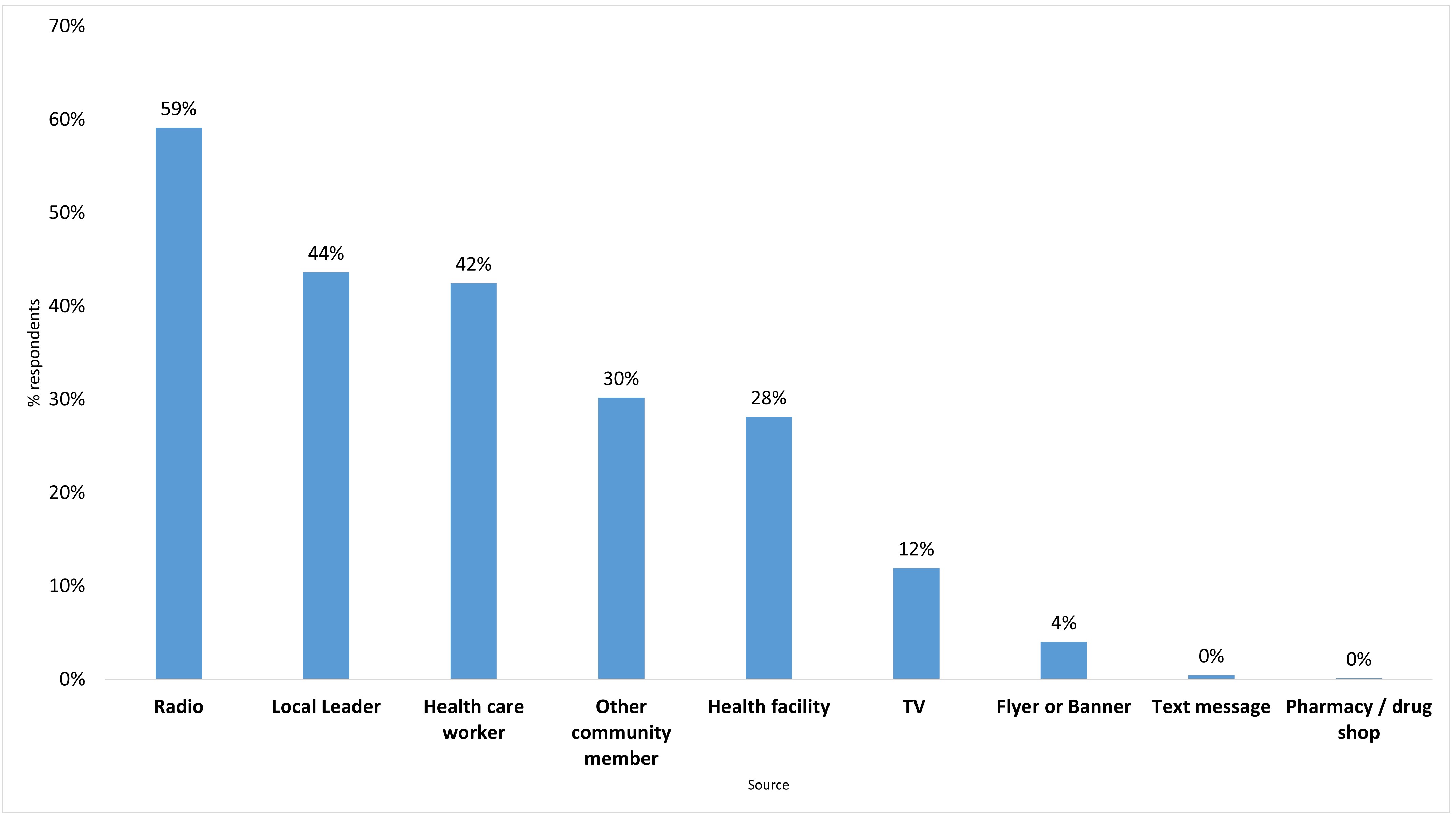

Supplement: Supplementary file 2 — Additional file 2. The sources of information on COVID-19. [file 12879_2023_8462_MOESM2_ESM.jpg]
