# Supplementary material for: Cholera and COVID-19 pandemic prevention in multiple hotspot districts of Uganda: vaccine coverage, adverse events following immunization and WASH conditions survey
Source: BMC Infect Dis. 2023 Jul 21;23:487. doi: 10.1186/s12879-023-08462-y (PMC10362646; doi:10.1186/s12879-023-08462-y)
Supplement: Supplementary file 3 — Additional file 3. Percent of households that boiled or treated drinking water by district and educational level in the six study districts of Uganda. [file 12879_2023_8462_MOESM3_ESM.pdf]

Table 3.1: Percent of households that boiled or treated drinking water by district and educational level in the study area

Background characteristics	Yes, boiled, % (95%CI)	Yes, treated, % (95%CI)	Both boiled and treated, % (95%CI)	Nether boiled nor treated, % (95%CI)	Number of households (n)
**Overall**	**25.7 (22.8-28.7)**	**11.1(9.1-13.3)**	**2.7 (1.7-3.9)**	**60.6 (57.3-63.8)**	**900**
**District**					
Busia	55.3 (47.0-63.4)	6.7 (3.2-11.9)	6.0 (2.8-11.1)	32.0 (24.6-40.1)	150
Kasese	22.7 (16.2-30.2)	16.7 (11.1-23.6)	2.7 (0.7-6.7)	58.0 (49.7-66.0)	150
Madi-Okollo	6.7 (3.2-11.9)	4.7 (1.9-9.4)	0.0	88.7 (82.5-93.3)	150
Namayingo	22.7 (16.2-30.2)	18.0 (12.2-25.1)	1.3 (0.16-4.7)	58.0 (49.7-66.0)	150
Ntoroko	36.7 (29.0-44.9)	9.3 (5.2-15.2)	4.0 (1.5-8.5)	50.0 (41.7-58.3)	150
Obongi	10.0 (5.7-16.0)	11.3 (6.7-17.5)	2.0 (0.4-5.7)	76.7 (69.1-83.2)	150
**Education**					
No formal education	15.3 (9.1-22.7)	11.9 (6.5-18.8)	0.8 (0.0-4.5)	72.0 (62.7-79.5)	120
Primary	23.0 (19.3-27.1)	11.8 (9.1-15.1)	1.5 (0.6-3.0)	63.6 (59.1-68.0)	473
Secondary	30.8 (25.0-37.1)	11.8 (8.0-16.6)	5.1 (2.6-8.7)	55.7 (49.1-62.1)	237
Post-Secondary	44.3 (32.4-56.7)	11.8 (5.1-21.3)	5.7 (1.6-14.0)	35.7 (24.6-48.1)	70

The difference in boiling of water between those with low level of water boiling and that of Busia district (relatively high, above 50%) was statistically significant. There was variation in boiling of drinking water with education level. The higher the education the more likely the respondents were to boil the drinking water.
